# Comparison of classification methods for detecting associations between SNPs and chick mortality

**DOI:** 10.1186/1297-9686-41-18

**Published:** 2009-01-23

**Authors:** Nanye Long, Daniel Gianola, Guilherme JM Rosa, Kent A Weigel, Santiago Avendaño

**Affiliations:** 1 Department of Animal Sciences, University of Wisconsin, Madison, WI 53706, USA; 2 Department of Dairy Science, University of Wisconsin, Madison, WI 53706, USA; 3 Aviagen Ltd., Newbridge, Midlothian, EH28 8SZ, UK

## Abstract

Multi-category classification methods were used to detect SNP-mortality associations in broilers. The objective was to select a subset of whole genome SNPs associated with chick mortality. This was done by categorizing mortality rates and using a filter-wrapper feature selection procedure in each of the classification methods evaluated. Different numbers of categories (2, 3, 4, 5 and 10) and three classification algorithms (naïve Bayes classifiers, Bayesian networks and neural networks) were compared, using early and late chick mortality rates in low and high hygiene environments. Evaluation of SNPs selected by each classification method was done by predicted residual sum of squares and a significance test-related metric. A naïve Bayes classifier, coupled with discretization into two or three categories generated the SNP subset with greatest predictive ability. Further, an alternative categorization scheme, which used only two extreme portions of the empirical distribution of mortality rates, was considered. This scheme selected SNPs with greater predictive ability than those chosen by the methods described previously. Use of extreme samples seems to enhance the ability of feature selection procedures to select influential SNPs in genetic association studies.

## Introduction

In genetic association studies of complex traits, assessing many loci jointly may be more informative than testing associations at individual markers. Firstly, the complexity of biological processes underlying a complex trait makes it probable that many loci residing on different chromosomes are involved [1,2]. Secondly, carrying out thousands of dependent single marker tests tends to produce many false positives. Even when significance thresholds are stringent, "significant" markers that are detected sometimes explain less than 1% of the phenotypic variation [3].

Standard regression models have problems when fitting effects of a much larger number of SNPs (and, possibly, their interactions) than the number of observations available. To address this difficulty, a reasonable solution could be pre-selection of a small number of SNPs, followed by modeling of associations between these SNPs and the phenotype [4]. Other strategies include stepwise selection [5], Bayesian shrinkage methods [6], and semiparametric procedures, such as mixed models with kernel regressions [7,8].

Machine learning methods are alternatives to traditional statistical approaches. Machine learning is a branch of artificial intelligence that "learns" from past examples, and then uses the learned rules to classify new data [9]. Their typical use is in a classification framework, *e.g*., disease classification. For example, Sebastiani *et al*. [10] applied Bayesian networks to predict strokes using SNP information, as well as to uncover complex relationships between diseases and genetic variants. Typically, classification is into two classes, such as "unaffected" and "affected". Multi-category classification has been studied, for example, by Khan *et al*. [11] and Li *et al*. [12]. It is more difficult than binary assignment, and classification accuracy drops as the number of categories increases. For instance, the error rate of random classification is 50% and 90% when 2 and 10 categories are used, respectively.

In a previous study of SNP-mortality association in broilers [13], the problem was cast as a case-control binary classification by assigning sires in the upper and lower tails of the empirical mortality rate distribution, into high or low mortality classes. Arguably, there was a loss of information about the distribution, because intermediate sires were not used. In the present work, SNP-mortality associations were studied as a multi-category classification problem, followed by a filter-wrapper SNP selection procedure [13] and SNP evaluations. All sire family mortality rates were classified into specific categories based on their phenotypes, and the number of categories was varied (2, 3, 4, 5 or 10). The objectives were: 1) to choose an integrated SNP selection technique by comparing three classification algorithms, naïve Bayes classifier (NB), Bayesian network (BN) and neural network (NN), with different numbers of categories, and 2) to ascertain the most appropriate use of the sire samples available.

## Methods

### Data

Genotypes and phenotypes came from the Genomics Initiative Project at Aviagen Ltd. (Newbridge, Scotland, UK). Phenotypes consisted of early (0–14d) and late (14–42d) age mortality status (dead or alive) of 333,483 chicks. Birds were raised in either high (H) or low (L) hygiene conditions: 251,539 birds in the H environment and 81,944 in the L environment. The H and L environments were representative of those in selection nucleus and commercial levels, respectively, in broiler breeding. Information included sire, dam, dam's age, hatch and sex of each bird. There were 5,523 SNPs genotyped on 253 sires. Each SNP was bi-allelic (*e.g*., "A" or "G" alleles) and genotypes were arbitrarily coded as 0 (AA), 1 (AG) or 2 (GG). A detailed description of these SNPs is given in Long *et al*. [13].

The entire data set was divided into four strata, each representing an age-hygiene environment combination. For example, records of early mortality status of birds raised in low hygiene conditions formed one stratum, denoted as EL (early age-low hygiene). Similarly, the other three strata were EH (early age-high hygiene), LL (late age-low hygiene) and LH (late age-high hygiene). Adjusted sire mortality means were constructed by fitting a generalized linear mixed model (with fixed effect of dam's age and random effect of hatch) to data (dead or alive) from individual birds, to get a residual for each bird, and then averaging progeny residuals for each sire (see Appendix). After removing SNPs with missing values, the numbers of sires and SNPs genotyped per sire were: EL and LL: 222 sires and 5,119 SNPs; EH and LH: 232 sires and 5,166 SNPs. Means and standard deviations (in parentheses) of adjusted sire means were 0.0021 (0.051), -0.00021 (0.033), -0.0058 (0.058) and 0.00027 (0.049) for EL, EH, LL and LH, respectively. Subsequently, SNP selection and evaluation were carried out in each of the four strata in the same way.

### Categorization of adjusted sire mortality means

Sire mortality means were categorized into *K *classes (*K *= 2, 3, 4, 5 or 10). The adjusted sire means were ordered, and each was assigned to one of *K *equal-sized classes in order to keep a balance between sizes of training samples falling into each category. For example, with *K *= 3, the thresholds determining categories were the 1/3 and 2/3 quantiles of the empirical distribution of sire means. This is just one of the many possible forms of categorization, and it does not make assumptions about the form of the distribution.

### "Filter-wrapper" SNP selection

A two-step feature selection method, "filter-wrapper", described in Long *et al*. [13] was used. There, upper and lower tails of the distribution of sire means were used as case-control samples, and the classification algorithm used in the wrapper step was naïve Bayes. In the present study, all sires were used in a multi-category classification problem, and three classification algorithms (NB, BN and NN) were compared.

#### "Filter" step

A collection of 50 "informative" SNPs was chosen in this step. It was based on information gain [9], a measure of how strongly a SNP is associated with the category distinction of sire mortality means. Briefly, information gain is the difference between entropy of the mortality rate distribution before and after observing the genotype at a given SNP locus. The larger the information gain, the more the SNP reduces uncertainty about mortality rate. As noted earlier, the 50 top scoring SNPs with respect to their information gain were retained for further optimization in the wrapper step. The filter procedure was coded in Java.

#### "Wrapper" step

This procedure is an iterative search-and-evaluate process, using a specific classification algorithm to evaluate a subset of SNPs (relative to the full set of 50 SNPs) searched [14]. Three classification algorithms, NB, BN and NN, were compared in terms of the cross-validation classification accuracy of the chosen subset of SNPs. Two widely used search methods are forward selection (FS) and backward elimination (BE) [15]. FS starts from an empty set and progressively adds SNPs one at time; BE starts with the full set, and removes SNPs one at a time. The search methods stop when there is no further improvement in classification accuracy. In general, BE produces larger SNP sets and better classification accuracy than FS [13,16], but it is more time-consuming. Differences in computation time between BE and FS were large when the classification algorithm was BN, which was computationally intensive. However, the difference between FS and BE in terms of classification accuracies of the chosen SNP subsets was small (Appendix). Hence, FS was adopted for BN. For NB and NN the search method was BE. The wrapper procedure was carried out on the Weka platform [16]. Computing time for running wrapper using the search method selected for each of NB, BN and NN was 1 min for NB, 3 min for BN and 8.2 h for NN. These were benchmarked on a dataset with 222 sires and 50 SNPs, which was typical for each stratum.

#### Naïve Bayes

**Figure 1:**
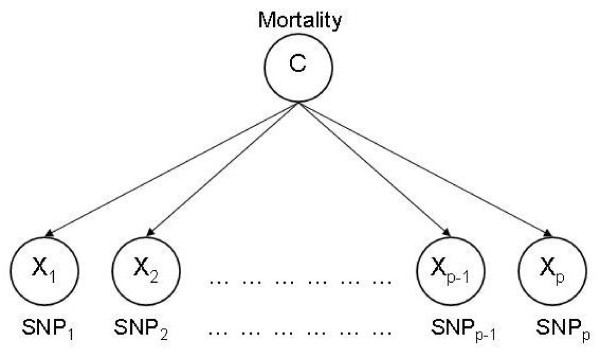
**Illustration of naïve Bayes (NB)**. *X*_1_,..., *X*_*p *_are SNPs used to predict class *C *(*e.g*., "low" or "high" mortality). NB assumes SNP independence given *C*.

Let (*X*_1_,..., *X*_*p*_) be features (SNPs) with discrete values (*e.g*., AA, AG or GG at a locus) used to predict class *C *("low" or "high" mortality). A schematic is in Figure 1. Given a sire with genotype (*x*_1_,..., *x*_*p*_), the best prediction of the mortality class to which it belongs is that given by class *c *which maximizes Pr(*C *= *c *| *X*_1 _= *x*_1_,..., *X*_*p *_= *x*_*p*_). By Bayes' theorem,

$$\mathrm{Pr}(C=c|X_{1}=x_{1},\ldots ,X_{p}=x_{p})=\frac{\mathrm{Pr}(X_{1}=x_{1},\ldots ,X_{p}=x_{p}|C=c)\mathrm{Pr}(C=c)}{\mathrm{Pr}(X_{1}=x_{1},\ldots ,X_{p}=x_{p})}.$$

Pr(*C *= *c*) can be estimated from training data and Pr(*X*_1 _= *x*_1_,..., *X*_*p *_= *x*_*p*_) is irrelevant for class allocation; the predicted value is the class that maximizes Pr(*X*_1 _= *x*_1_,..., *X*_*p *_= *x*_*p *_| *C *= *c*). NB assumes that *X*_1_,..., *X*_*p *_are conditionally independent given *C*, so that Pr(*X*_1 _= *x*_1_,..., *X*_*p *_= *x*_*p *_| *C *= *c*) can be decomposed as Pr(*X*_1 _= *x*_1 _| *C = c*) × ⋯ × Pr(*X*_*p *_= *x*_*p *_| *C = c*). Although the strong assumption of feature independence given class is often violated, NB often exhibits good performance when applied to data sets from various domains, including those with dependent features [17,18]. The probabilities, *e.g*., Pr(*X*_1 _= *x*_1 _| *C = c*), are estimated using the ratio between the number of sires with genotype *x*_1 _that are in class *c*, and the total number of sires in class *c*.

#### Bayesian networks

**Figure 2:**
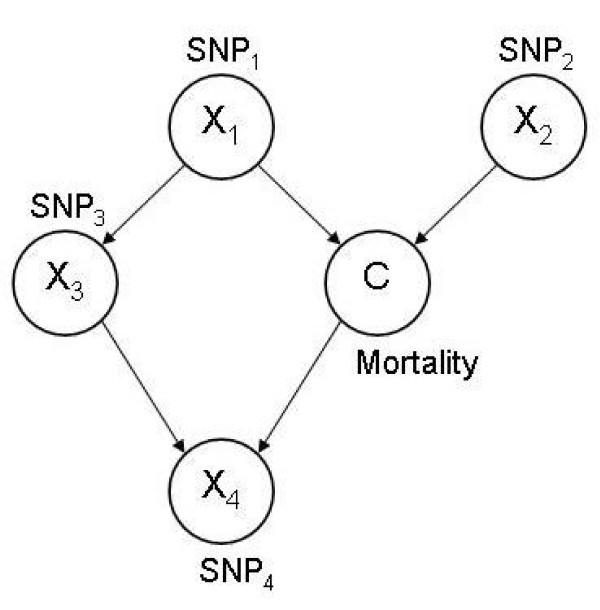
**Illustration of Bayesian networks (BN)**. Four nodes (*X*_1 _to *X*_4_) represent SNPs and one (*C*) corresponds to the mortality phenotype. Arrows between nodes indicate dependency.

Bayesian networks are directed acyclic graphs for representing probabilistic relationships between random variables [19]. Most applications of BN in genotype-phenotype association studies are in the context of case-control designs (*e.g*., [10]). A node in the network can represent a categorical phenotype (*e.g*., "low" or "high" mortality), and the other nodes represent SNPs or covariates, as illustrated in Figure 2 for a 5-variable network. To predict phenotype (*C*) given its "parent" nodes (Pa(*C*)) (*i.e*., nodes that point to *C*, such as SNP_1 _and SNP_2_), one chooses *c *which maximizes Pr(*C *= *c *| Pa(*C*)) [20]. To learn (fit) a BN, a scoring function that evaluates each network is used, and the search for an optimal network is guided by this score [21]. In this study, the scoring metric used was the Bayesian metric [22], which is the posterior probability of the network (*M*) given data (*D*): Pr(*M*|*D*)∝ Pr(*M*)Pr(*D*|*M*) Given a network *M*, if Dirichlet distributions are used as conjugate priors for parameters *θ*_*M *_(a vector of probabilities) in *M*, then, Pr(*D *| *M*) = ∫ Pr(*D*|θ_*M*_)Pr(θ_*M*_)*dθ*_*M*_, has a closed form. The search method used for learning *M *was a hill-climbing one, which considered arrow addition, deletion and reversal during the learning process [16]. This search-evaluation process terminated when there was no further improvement of the score. All networks were equally likely, a priori.

#### Neural networks

**Figure 3:**
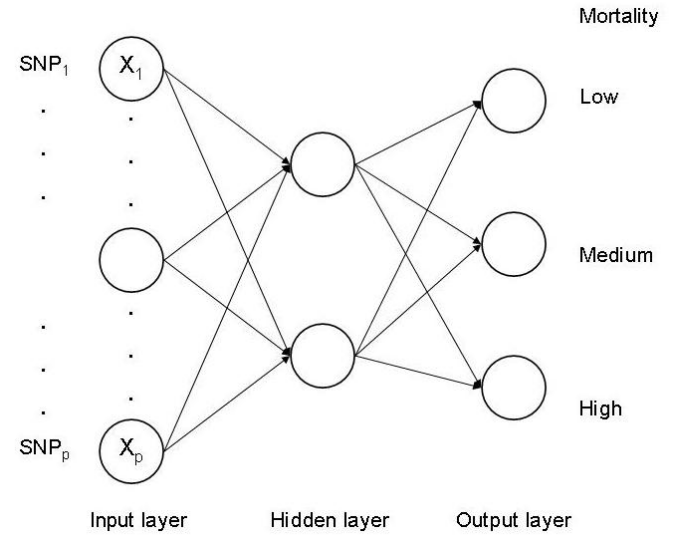
**Illustration of neural networks (NN)**. Each SNP occupies an input node and takes value 0, 1 or 2. The hidden nodes receive a weighted sum of inputs and apply an activation function to the sum. The output nodes then receive a weighted sum of the hidden nodes' outputs and, again, apply an activation function to the sum. For a 3-category classification (*K *= 3), three separate output nodes were used, with each node being specific to one category (low, medium or high). Classification was assigned to the category with the largest output value.

A neural network is composed of a set of highly interconnected nodes, and is a type of non-parametric regression approach for modeling complex functions [23,24]. The network used in this study, shown in Figure 3, is a 3-layer feedforward neural network. It contains an input layer, a hidden layer and an output layer. Each connection has an unknown weight associated with it, which determines the strength and sign of the connection. The input nodes are analogous to predictor variables in regression analysis; each SNP occupies an input node and takes value 0, 1 or 2. The hidden layer fitted contained two nodes, each node taking a weighted sum of all input nodes. The node was activated using the sigmoid function: $g(z_{h})={\left(1+e^{-z_{h}}\right)}^{-1}$, where $z_{h}=\sum_{j}w_{jh}x_{j}$; *x*_*j *_is SNP_*j *_and *w*_*jh *_is the weight applied to connection from SNP_*j *_to hidden node *h *(*h *= 1, 2). Similarly, a node in the output layer takes a weighted sum of all hidden nodes and, again, applies an activation function, and takes its value as the output of that node. The sigmoid function ranges from 0 to 1, and has the advantage of being differentiable, which is required for use in the back-propagation algorithm adopted in this study for learning the weights from inputs to hidden nodes, and from these to the output nodes [23]. For a *K*-category classification problem with continuous outputs (as per the sigmoid function), *K *output nodes were used, with each node being specific to one mortality category. Classification was assigned to the category with the largest output value. The back-propagation algorithm (a non-linear least-squares minimization) processes observation by observation, and it was iterated 300 times. The number of parameters in the network is equal to (*K *+ *M*) +*M *(*K *+ *N*), where *N*, *M *and *K *denote the number of nodes in the input, hidden and output layers, respectively. For example, in a binary classification (*K *= 2) with 50 input nodes representing 50 SNPs, and two hidden nodes, the number of parameters is 108.

### SNP subset evaluation

Comparison of the three classification algorithms (NB, BN and NN) yielded a best algorithm in terms of classification accuracy. Using the best classification algorithm, there were five optimum SNP subsets selected in the wrapper step in each stratum, corresponding to the 2, 3, 4, 5 or 10-category classification situation, respectively. The SNP subset evaluation refers to comparing the five best SNP subsets in a certain stratum (EL, EH, LL or LH). Two measures were used as criteria; one was the cross-validation predicted residual sum of squares (PRESS), and the other was the proportion of significant SNPs. In what follows, the two measures are denoted as A and B. Briefly, for measure A, a smaller value indicates a better subset; for measure B, a larger value indicates a better subset.

#### Measure A

PRESS is described in Ruppert *et al*. [25]. It is cross-validation based, and is related to the linear model:

$$M_{i}=\mu +\sum_{j=1}^{n_{g}}{\text{SNP}}_{ij}+e_{i},$$

where *M*_*i *_was sire *i*'s adjusted mortality mean (after standardization, to achieve a zero mean and unit variance); SNP_*ij *_denotes the fixed effect of genotype of SNP_*j *_in sire *i*; and *n*_*g *_is the number of SNPs in the subset under consideration. Although the wrapper selected a "team" of SNPs that act jointly, only their main effects were fitted for PRESS evaluation (to avoid running out of degrees of freedom). The model was fitted by weighted least squares, with the weight for a sire family equal to the proportion of progeny contributed by this sire. The errors were assumed to have a Student-t distribution with 8 degrees of freedom (*t-*8) distribution, after examining Q-Q plots with normal, *t*-4, *t*-6 and *t*-8 distributions. Given this model, PRESS was computed by

$$\text{PRESS}=\sum_{i=1}^{N}{\left(M_{i}-{\hat{M}}_{\left(i\right)}\right)}^{2}.$$

Here, *M*_*i *_is predicted using all sire means except the *i*th (*i *= 1, 2,..., *N*) sire, and this predicted mean is denoted by ${\hat{M}}_{\left(i\right)}$. A subset of SNPs was considered "best" if it produced the smallest PRESS when employing this subset as predictors. A SAS^® ^macro was written to generate PRESS statistics and it was embedded in SAS^® ^PROC GLIMMIX (SAS^® ^9.1.3, SAS^® ^Institute Inc., Cary, NC).

#### Measure B

This procedure involved calculating how many SNPs in a subset were significantly associated with the mortality phenotype. Given a subset of SNPs, an *F*-statistic (in the ANOVA sense) was computed for each SNP. Subsequently, given an individual SNP's *F-*statistic, its *p*-value was approximated by shuffling phenotypes across all sires 200 times, while keeping the sires' genotypes for this SNP fixed. Then, the proportion of the 200 replicate samples in which a particular *F*-statistic exceeded that of the original sample was calculated. This proportion was taken as the SNP's *p*-value. After obtaining *p*-values for all SNPs in the subset, significant SNPs were chosen by controlling the false discovery rate at level 0.05 [26]. The proportion of significant SNPs in a subset was the end-point.

### Comparison of using extreme sires vs. using all sires

This comparison addressed whether or not the loss of information from using only two extreme tails of the sample, as in Long *et al*. [13], affected the "goodness" of the SNP subset selected. Therefore, SNP selection was also performed by an alternative categorization method based on using only two extreme portions of the entire sample of sire means. The two thresholds used were determined by α, such that one was the 100 × α% quantile of the distribution of sire mortality means, and the other was the 100×(1-α)% quantile. SNP selection was based on the filter-wrapper method, as for the multi-category classification, with NB adopted in the wrapper step. Four α values, 0.05, 0.20, 0.35 and 0.50, were considered, and each yielded one partition of sire samples and, correspondingly, one selected SNP subset.

In each situation (using all sires *vs*. extreme sires only), the best subset was chosen by the PRESS criterion, as well as by its significance level. That is, the smallest PRESS was selected as long as it was significant at a predefined level (e.g., *p *= 0.01); otherwise, the second smallest PRESS was examined. This guaranteed that PRESS values of the best SNP subsets were not obtained by chance. Significance level of an observed PRESS statistic was assessed by shuffling phenotypes across all sires 1000 times, while keeping unchanged sires' genotypes at the set of SNPs under consideration. This procedure broke the association between SNPs and phenotype, if any, and produced a distribution of PRESS values under the hypothesis of no association. The proportion of the 1000 permutation samples with smaller PRESS than the observed one was taken as its *p*-value.

## Results

### Comparison of NB, BN and NN

Classification error rates (using 10-fold cross-validation) of the final SNP subsets selected by the "wrapper" with the three classification algorithms are in Table 1. As expected, error rates increased with *K *for each classifier, since the baseline error increased with *K*; in each instance, classifiers improved upon random classification. In all cases, NB had the smallest error rates, and by a large margin. For example, with *K *= 2, error rates of NB were about half of those achieved with either BN or NN. Therefore, NB was used for further analysis.

**Table 1:** Classification error rates using naïve Bayes (NB), Bayesian networks (BN) and neural networks (NN) in five categorization schemes (*K *= 2, 3, 4, 5 and 10), based on the final SNP subsets selected

		Number of categories (*K*)				
		
Stratum^a^	Classifier	*K *= 2	*K *= 3	*K *= 4	*K *= 5	*K *= 10
EL	NB	0.124	0.225	0.314	0.329	0.523
	BN	0.207	0.437	0.649	0.674	0.813
	NN	0.270	0.295	0.543	0.662	0.813
						
EH	NB	0.116	0.212	0.330	0.397	0.506
	BN	0.228	0.422	0.653	0.688	0.820
	NN	0.185	0.364	0.560	0.623	0.827
						
LL	NB	0.132	0.221	0.375	0.408	0.523
	BN	0.225	0.403	0.545	0.709	0.824
	NN	0.221	0.401	0.588	0.610	0.831
						
LH	NB	0.151	0.252	0.338	0.405	0.494
	BN	0.261	0.438	0.532	0.681	0.816
	NN	0.278	0.381	0.530	0.534	0.793

^a ^EL = early age-low hygiene; EH = early age-high hygiene; LL = late age-low hygiene; LH = late age-high hygiene.

### Evaluation of SNP subsets

Results of the comparison of the five categorization schemes (*K *= 2, 3, 4, 5 and 10) using measures A and B are shown in Table 2. The approach favored by the two measures was typically different. For EL, measures A (PRESS) and B (proportion of significant SNPs) agreed on *K *= 2 as best. For EH, *K *= 2 and *K *= 3 were similar when using measure A; *K *= 3 was much better than the others when using method B. For LL, *K *= 2 was best for measure A whereas *K *= 3 or 4 was chosen by B. For LH, *K *= 3 and *K *= 2 were best for measures A and B, respectively. Overall, classification with 2 or 3 categories was better than classification with more than 3 categories. This implies that measures A and B were not improved by using a finer grading of mortality rates.

**Table 2:** Evaluating SNP subsets using predicted residual sum of squares (A) and proportion of significant SNPs (B)

		Number of categories (*K*)				
		
Stratum^a^	Measure	*K *= 2	*K *= 3	*K *= 4	*K *= 5	*K *= 10
EL	A	**0.672**	0.781	0.747	0.807	0.964
	B	**0.53**	0.47	0.52	0.42	0.52
						
EH	A	**0.377**	0.378	0.490	0.444	0.624
	B	0.03	**0.16**	0.05	0.02	0.05
						
LL	A	**0.519**	0.534	0.591	0.552	0.608
	B	0	0.33	**0.34**	0.05	0.12
						
LH	A	0.547	**0.470**	0.605	0.655	0.642
	B	**0.27**	0.24	0.15	0.25	0.23

^a ^EL = early age-low hygiene; EH = early age-high hygiene; LL = late age-low hygiene; LH = late age-high hygiene.

Best one among five SNP subsets according to measure A or B is in boldface.

SNP subsets selected under the five categorization schemes were compared with each other, to see if there were common ones. This led to a total of 10 pair-wise comparisons. The numbers of SNPs in these subsets differed, but were all less than 50, the full set size for "wrapper". As a result, the number of common SNPs ranged from 5 to 14 for stratum EL, 2 to 9 for EH, 2 to 13 for LH and 7 to 16 for LL.

### Comparison of using extreme sires vs. using all sires

**Figure 4:**
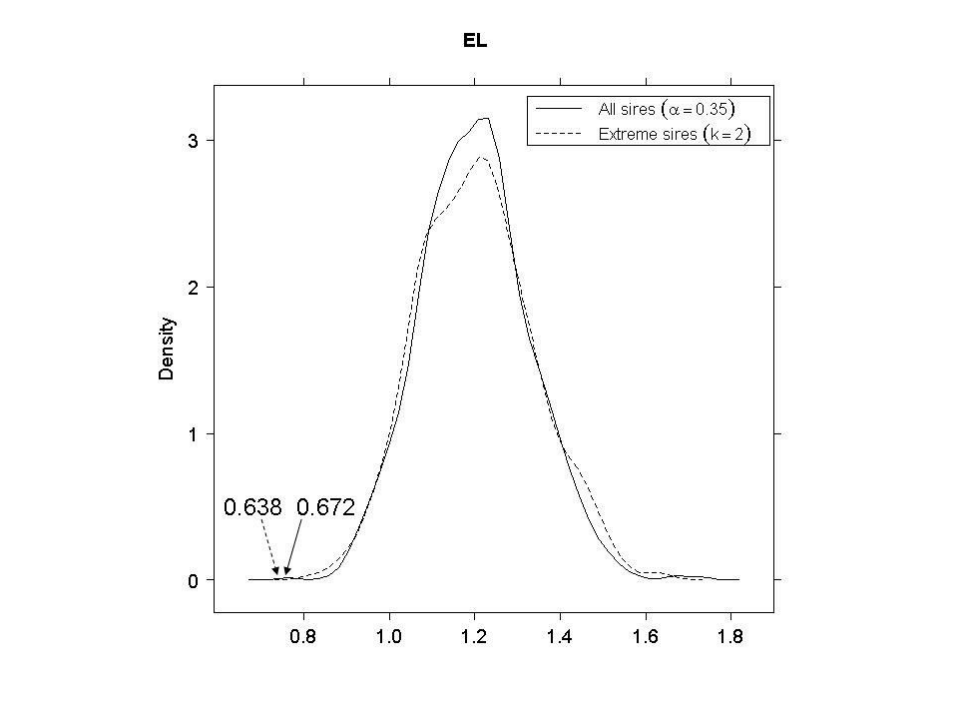
**Permutation distributions (1000 replicates) of predicted residual sum of squares (PRESS), for each of the four strata (part 1)**. (EL: early age-low hygiene, EH: early age-high hygiene, LL: late age-low hygiene and LH: late age-high hygiene). Observed PRESS values are marked in the plots, with dashed arrows when using extreme sires and solid arrows when using all sires.

**Figure 5:**
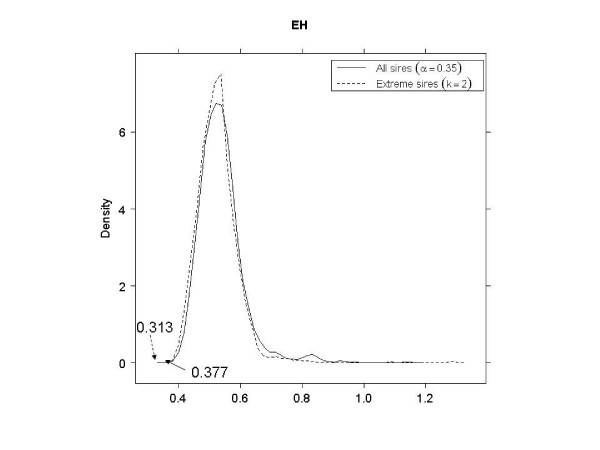
**Permutation distributions (1000 replicates) of predicted residual sum of squares (PRESS), for each of the four strata (part 2)**. (EL: early age-low hygiene, EH: early age-high hygiene, LL: late age-low hygiene and LH: late age-high hygiene). Observed PRESS values are marked in the plots, with dashed arrows when using extreme sires and solid arrows when using all sires.

**Figure 6:**
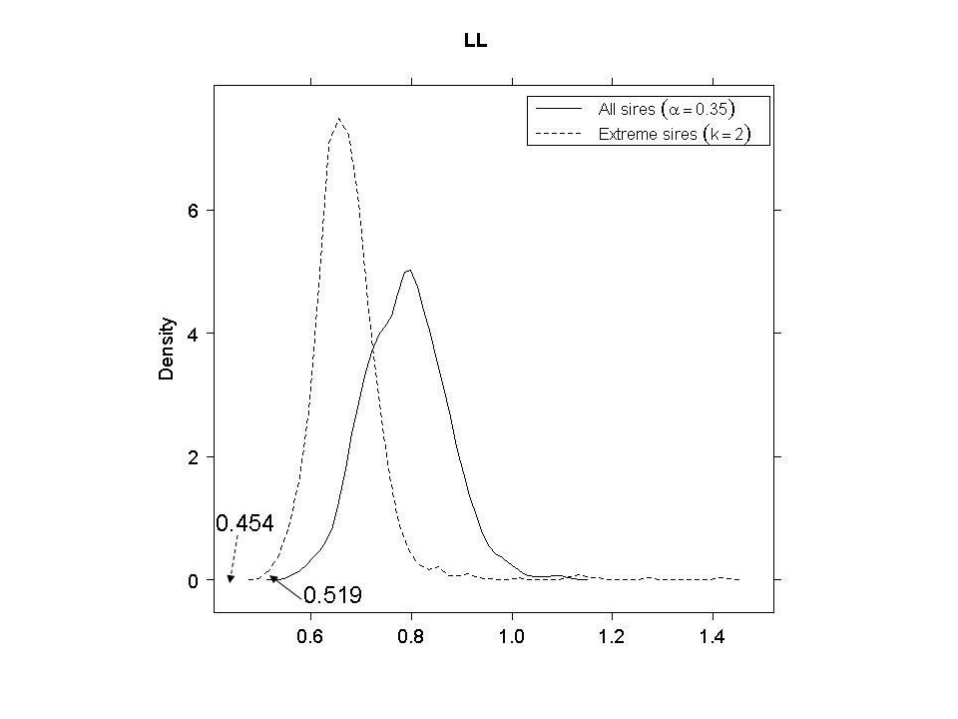
**Permutation distributions (1000 replicates) of predicted residual sum of squares (PRESS), for each of the four strata (part 3)**. (EL: early age-low hygiene, EH: early age-high hygiene, LL: late age-low hygiene and LH: late age-high hygiene). Observed PRESS values are marked in the plots, with dashed arrows when using extreme sires and solid arrows when using all sires.

**Figure 7:**
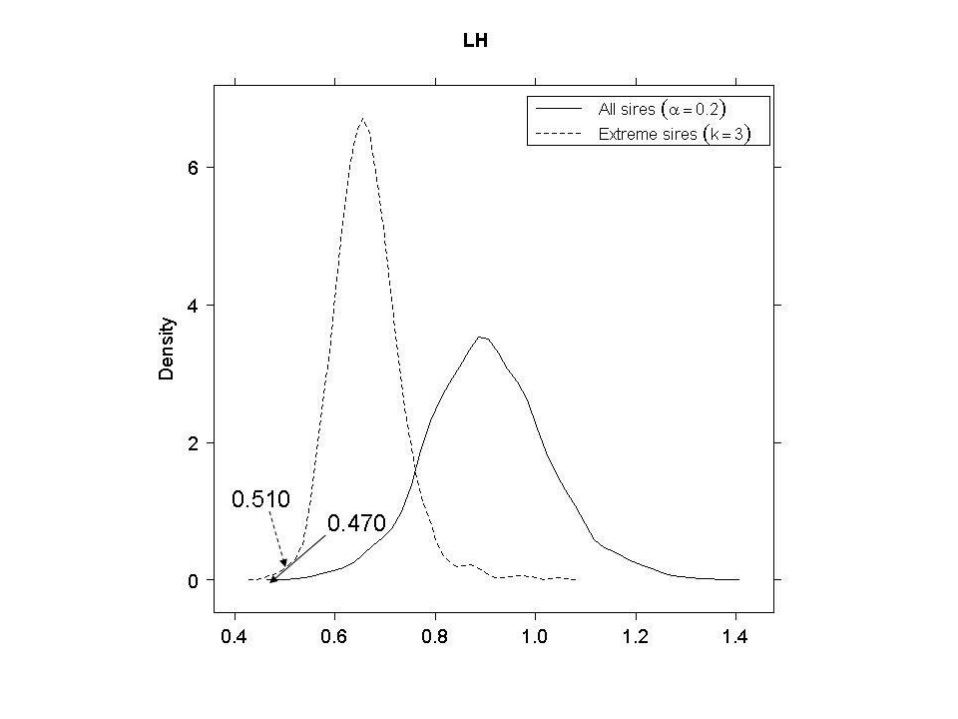
**Permutation distributions (1000 replicates) of predicted residual sum of squares (PRESS), for each of the four strata (part 4)**. (EL: early age-low hygiene, EH: early age-high hygiene, LL: late age-low hygiene and LH: late age-high hygiene). Observed PRESS values are marked in the plots, with dashed arrows when using extreme sires and solid arrows when using all sires.

As shown in Table 3, in EL, EH and LL, better SNP subsets (smaller PRESS values) were obtained when using the tails of the distribution of sires, as opposed to using all sires. In LH, a 3-category classification using all sires had a smaller PRESS than a binary classification using 40% of the sire means. In LH with extreme sires, the smallest PRESS value (0.498) was not significant (*p *= 0.915). This was possibly due to the very small size of the corresponding SNP subset; there were only four SNPs with 3^4 ^= 81 genotypes, so the observed PRESS would appear often in the null distribution. Therefore, the second smallest PRESS value (0.510) was used to compare against using all sires. Figures 4, 5, 6 and 7 shows the null distributions (based on 1000 permutations) of PRESS values when SNPs were selected using extreme sires or all sires in each stratum. All observed values were "significant" (*p *≤ 0.007), indicating that the PRESS of each SNP subset was probably not due to chance.

**Table 3:** Comparison of SNP selection using sires with extreme phenotypes *vs*. using all sires, in terms of predicted residual sum of squares of the best SNP subsets

	Extreme sires				All sires			
		
Stratum^a^	PRESS^b^	*p*-value^c^	α^d^	#SNPs^f^	PRESS^b^	*p*-value^c^	*K*^e^	#SNPs^f^
EL	0.638	0.001	0.35	34	0.672	0.001	2	36
EH	0.313	0.001	0.35	35	0.377	0.002	2	33
LL	0.454	0.001	0.35	29	0.519	0.001	2	46
LH	0.510	0.007	0.20	28	0.470	0.001	3	45

^a ^EL = early age-low hygiene; EH = early age-high hygiene; LL = late age-low hygiene; LH = late age-high hygiene.

^b ^predicted residual sum of squares.

^c ^*p*-value of PRESS, obtained from permutation distribution.

^d ^With extreme sires, two thresholds are determined by α.

^e ^With all sires, *K *is the number of categories.

^f ^Number of SNPs in the subset.

## Discussion

Arguably, the conditional independence assumption of NB, *i.e*., independence of SNPs given class, is often violated. However, it greatly simplifies the learning process, since the probabilities of each SNP genotype, given class, can be estimated separately. Here, NB clearly outperformed the two more elaborate methods (BN and NN). One reason could be that, although simple decomposition using the independence assumption results in poor estimates of Pr(*C *= *c *| *X*_1 _= *x*_1_,..., *X*_*p *_= *x*_*p*_), the correct class still has the highest estimated probability, leading to high classification accuracy of NB [17]. Another reason might be overfitting in BN and NN, especially in the current study, where there were slightly over 200 sires in total. Overfitting can lead to imprecise estimates of coefficients in NN, and imprecise inference about network structure and associated probabilities in BN. In this sense, a simpler algorithm, such as NB, seems more robust to noisy data than complex models, since the latter may fit the noise. The best way to avoid overfitting is to increase size of training data, so that it is sufficiently large relative to the number of model parameters (*e.g*., 5 times as many training cases as parameters). If sample size is fixed, approaches for reducing model complexity have to be used. In the case of NN, one can reduce the number of hidden nodes or use regularization (weight decay), to control magnitude of weights [27]. For BN, the number of parent nodes for each node can be limited in advance, to reduce the number of conditional probability distributions involved in the network. One can also choose a network quality measure that contains a penalty for network size, for example, the Bayesian information criterion [28] and the minimal description length [29]. These measures trade off "goodness-of-fit" with complexity of the model. Finally, one may consider other classifiers that are less prone to overfitting, such as support vector machines (SVMs) [30]. Guyon *et al*. [31] presented a recursive feature elimination-based SVM (SVM-RFE) method for selecting discriminant genes, by using the weights of a SVM classifier to rank genes. Unlike ranking which is based on individual gene's relevance, SVM-RFE ranking is a gene subset ranking and takes into account complementary relationship between genes.

An alternative to the filter-wrapper approach for handling a large number of genetic markers is the random forests methodology [32], which uses ensembles of trees. Each tree is built on a bootstrap sample of the original training data. Within each tree, the best splitting SNP (predictor) at each node is chosen from a random set of all SNPs. For prediction, votes from each single tree are averaged. Random forests does not require a pre-selection step, and ranks SNPs by a variable importance measure, which is the difference in prediction accuracy before and after permuting a SNP. Unlike a univariate one-by-one screening method, which may miss SNPs with small main effects but large interaction effects, ranking in random forests takes into account each SNP's interaction with others. Thus, random forests have gained attention in large scale genetic association studies, for example, for selecting interacting SNPs [33]. In fact, the wrapper is designed to address the same problem, by evaluating a subset of SNPs rather than a single SNP at a time. However, it cannot accommodate the initial pool of a large number of SNPs due to computational burden, so a pre-selection stage is required. In this sense, wrapper is not as efficient as random forests. In the case when correlated predictors exist, Strobl *et al*. [34] pointed out that the variable importance measures used in ordinary random forests may lead to biased selection of non-influential predictors correlated to influential ones, and proposed a conditional permutation scheme that could better reflect the true importance of predictors.

The number of top scoring SNPs (50) was set based on a previous study [13], where it was found that, starting with different numbers (50, 100, 150, 200 and 250) of SNPs, a naïve Bayes wrapper led to similar classification performances. To reduce model complexity and to save computational time, a smaller number of SNPs is preferred. To examine whether the 50 SNPs were related to each other or not, a redundancy measure was computed, to measure similarity between all pairs of the 50 SNPs (1225 pairs in total). Redundancy is based on mutual information between two SNPs, and ranges from 0 to 0.5, as in Long *et al*. [13]. Redundancies were low and under 0.05 for almost all pairs. For example, in stratum EL-3-category classification, 1222 out of 1225 pairs had values under 0.05. This indicates that SNP colinearity was unlikely in the subsequent wrapper step, which involved training classifiers using the SNP inputs.

As illustrated by the error rates found in the present study, multi-category classification gets harder as the number of categories (*K*) increases. This is because the baseline predictive power decreases with *K*, and average sample size for each category also decreases with *K*, which makes the trained model less reliable. To make a fair comparison among SNP subsets found with different *K*, the same evaluation procedure, neutral with respect to filter-wrapper and *K*, was uniformly applied to each setting. By using mortality means in their original form (a continuous response variable, as opposed to a discretized variable), two measures were used. Measure A (PRESS) evaluated a subset of SNPs from the perspective of predictive ability, while measure B estimated the proportion of SNPs in a subset that had a statistically significant association with mortality. Although the best SNP subset was measure-dependent, it was either with *K *= 2 or *K *= 3. Thus, it appears that classification into two or three categories is sufficient.

The comparison between SNP subsets selected using sires with extreme phenotypic values and those selected using all sire means indicated better performance of the former strategy of SNP detection. This is so, at least in part, because concern is about classification accuracy, and obtaining more informative samples for each class is more important than avoiding loss of information resulting from discarding some sire means. Perhaps including all sire samples brings noise, leading to a poorer predictive ability of the selected SNPs. In order to assess significance of the observed difference in PRESS between using "extreme" and "all" strategies, one can shuffle sire means over genotypes B (*e.g*., 1000) times, generating B permutation samples. For each of the B samples, apply "extreme" and "all" to get PRESS, and then take their difference. This would produce a null distribution, against which the observed difference in PRESS can be referred to. This was not done in this study, due to extra computational intensiveness.

In the context of selecting SNPs associated with chick mortality, two conclusions emerge. First, if one wishes to utilize all sire samples available, a good choice consists of a naïve Bayes classifier, coupled with a categorization of mortality rates into two or three classes. Second, one may want to use more extreme (hence more representative) samples, even at the cost of losing some information, to achieve better predictive ability on the selected SNPs.

In summary, and in the spirit of the studies of Long *et al*. [13,35], a filter-wrapper two step feature selection method was used effectively to ascertain SNPs associated with quantitative traits. The sets of interacting SNPs identified in this procedure can then be used in statistical models for genomic-assisted prediction of quantitative traits [7,8,36-38].

## Competing interests

The authors declare that they have no competing interests.

## Authors' contributions

NL conceived, carried out the study and wrote the manuscript; DG conceived, supervised the study and wrote the manuscript; GR, KW and SA helped to coordinate the study and provided critical insights.

## Appendix

### Calculation of adjusted sire means by strata

To create a response variable for each of the sires genotyped, effects of factors (dam's age and hatch) potentially affecting individual bird survival were removed via a generalized linear mixed model (GLMM) without sire and hygiene effects. For each bird, a residual derived from the GLMM was calculated. Birds were classified into two groups, corresponding to the hygiene environments (L and H) in which they had been raised. In each hygiene group, residuals of progeny of a sire were averaged, producing an adjusted progeny mortality mean as the response variable for each sire.

The individual record on each bird was binary (dead or alive), and the GLMM fitted was:

(1) logit(*p*_*ijk*_) = *μ *+ DA_*i *_+ H_*j*_,

where *p*_*ijk *_is the death probability of bird *k*, progeny of a dam of age *i *and born in hatch *j*. Here, DA_*i *_stands for the fixed effect of the *i*th level of dam's age (*i *= 1, 2,..., 18); H_*j *_denotes the random effect of hatch *j *(*j *= 1, 2,..., 232), which was assumed normal, independent and identically distributed as *H*_*j*_~NIID(0, $\sigma_{H}^{2}$), where $\sigma_{H}^{2}$ was the variance between hatches. Let *y*_*ijk *_be the true binary status of bird *ijk *(0 = alive, 1 = dead) and ${\hat{p}}_{ijk}$ be the fitted death probability using model (1); then, the residual for a given bird is *r*_*ijk *_= *y*_*ijk *_- ${\hat{p}}_{ijk}$, with a sampling space of [-1,1]. GLMM was implemented in SAS^® ^PROC GLIMMIX (SAS^® ^9.1.3, SAS^® ^Institute Inc., Cary, NC).

Model (1) was fitted to both early and late mortality data. Subsequently, birds were divided into the two hygiene groups, and progeny residuals were averaged for each sire, as described above. Thus, four strata of age-hygiene combinations were formed, with each stratum containing the adjusted progeny mortality means calculated for: 1) birds of early age raised in low hygiene (EL); 2) birds of early age raised in high hygiene (EH); 3) birds of late age raised in low hygiene (LL); and 4) birds of late age raised in high hygiene (LH).

### Choice of search method for BN-based wrapper

**Figure 8:**
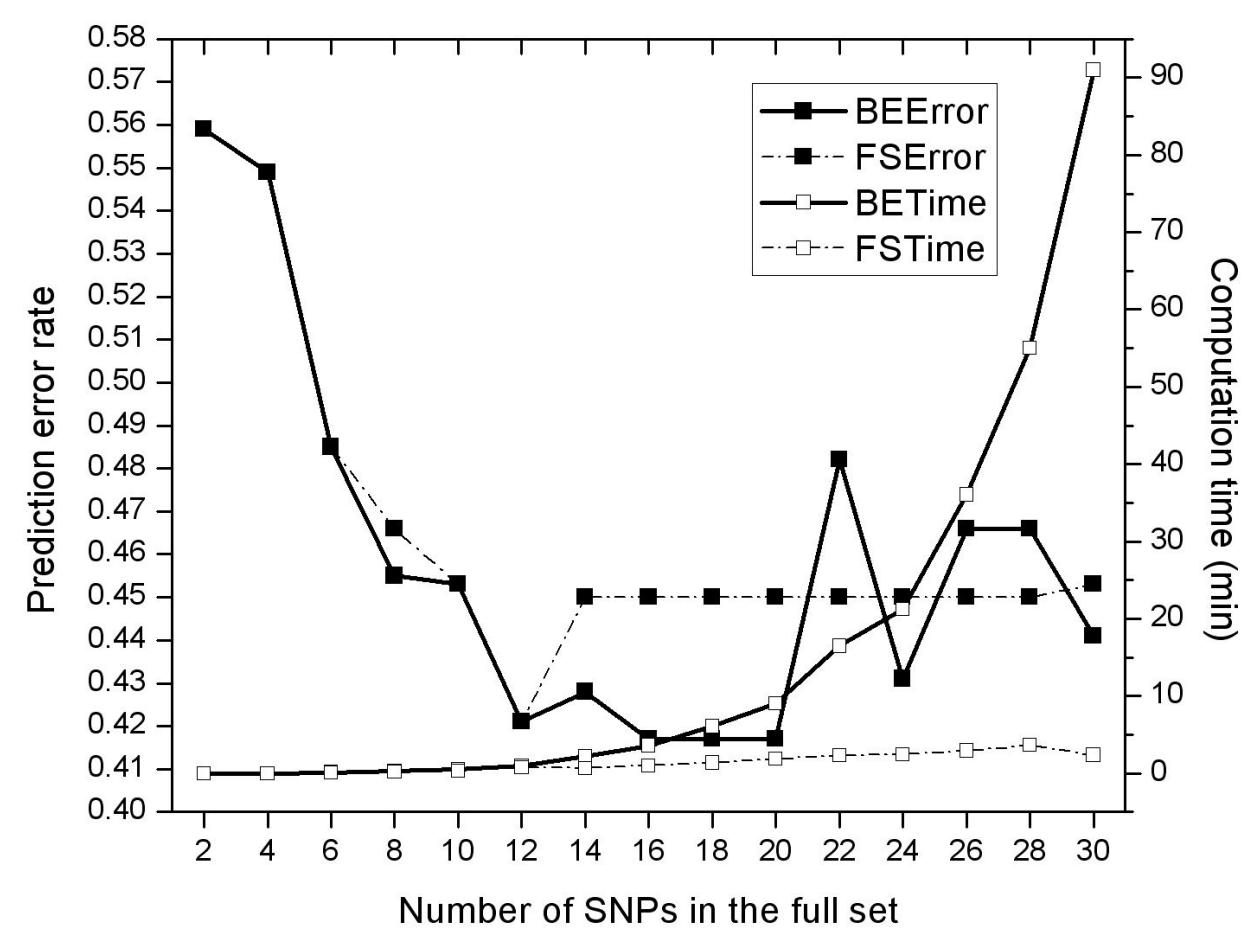
**Comparison of backward elimination (BE) and forward selection (FS) in the wrapper-based SNP subset selection**.

Choosing a proper search method was relevant primarily to a BN-wrapper, because of computational issues. Using data from the EL stratum and for *K *= 3 categories, computing time and predictive (classification) performance were monitored simultaneously for BE and FS (Fig 8). Computational time was the total time consumed by wrapper; prediction performance was measured as error rate of the final best SNP subset selected by wrapper. The full set's size was increased by 2 SNPs at a time until reaching 30. The difference between BE and FS in terms of computing time was clear (see two hollow-square lines): BE-time grew rapidly with the number of SNPs, while FS-time was lower and stable. The difference in time consumed was up to about 90 min for 30 SNPs. In contrast, the difference between BE and FS in terms of their prediction error rates was small. Patterns of Figure 8 should apply to other situations as well. The BN-wrapper adopted FS as search method in this work.

## References

[B1] Blakey JD Looking for a bit of co-action? Thorax 2007 62 196 197 17329556 10.1136/thx.2006.070649 PMC2117159

[B2] Dekkers JCM Hospital F The use of molecular genetics in the improvement of agricultural populations Nat Rev Genet 2002 3 22 32 11823788 10.1038/nrg701

[B3] Maier LM Howson JMM Walker N Spickett GP Jones RW Ring SM McArdle WL Lowe CE Bailey R Payne F Association of IL13 with total IgE: Evidence against an inverse association of atopy and diabetes J Allergy Clin Immunol 2006 117 1306 1313 16750991 10.1016/j.jaci.2005.12.1354

[B4] Hoh J Wille A Zee R Cheng S Reynolds R Lindpaintner K Ott J Selecting SNPs in two-stage analysis of disease association data: a model-free approach Ann Hum Genet 2000 64 413 417 11281279 10.1046/j.1469-1809.2000.6450413.x

[B5] Cordell HJ Clayton DG A unified stepwise regression procedure for evaluating the relative effects of polymorphisms within a gene using case/control or family data: application to HLA in type 1 diabetes Am J Hum Genet 2002 70 124 141 11719900 10.1086/338007 PMC384883

[B6] Wang H Zhang Y-M Li X Masinde GL Mohan S Baylink DJ Xu S Bayesian shrinkage estimation of quantitative trait loci parameters Genetics 2005 170 465 480 15781696 10.1534/genetics.104.039354 PMC1449727

[B7] Gianola D Fernando RL Stella A Genomic-assisted prediction of genetic value with semiparametric procedures Genetics 2006 173 1761 1776 16648593 10.1534/genetics.105.049510 PMC1526664

[B8] Gianola D van Kaam JBCHM Reproducing kernel Hilbert spaces regression methods for genomic assisted prediction of quantitative traits Genetics 2008 178 2289 2303 18430950 10.1534/genetics.107.084285 PMC2323816

[B9] Mitchell TM Machine Learning 1997 Hightstown, NJ: McGraw-Hill

[B10] Sebastiani P Ramoni MF Nolan V Baldwin CT Steinberg MH Genetic dissection and prognostic modeling of overt stroke in sickle cell anemia Nat Genet 2005 37 435 440 15778708 10.1038/ng1533 PMC2896308

[B11] Khan J Wei JS Ringner M Saal LH Ladanyi M Westermann F Berthold F Schwab M Antonescu CR Peterson C Meltzer PS Classification and diagnostic prediction of cancers using gene expression profiling and artificial neural networks Nat Med 2001 7 673 679 11385503 10.1038/89044 PMC1282521

[B12] Li T Zhang C Ogihara M A comparative study of feature selection and multiclass classification methods for tissue classification based on gene expression Bioinformatics 2004 20 2429 2437 15087314 10.1093/bioinformatics/bth267

[B13] Long N Gianola D Rosa GJM Weigel KA Avendano S Machine learning classification procedure for selecting SNPs in genomic selection: application to early mortality in broilers J Anim Breed Genet 2007 124 377 389 18076475 10.1111/j.1439-0388.2007.00694.x

[B14] Kohavi R John GH Wrappers for feature subset selection Artificial Intelligence 1997 97 273 324

[B15] Caruana R Freitag D Greedy attribute selection International Conference on Machine Learning 1994 28 36

[B16] Witten IH Frank E Data Mining: Practical Machine Learning Tools and Techniques 2005 San Francisco, CA: Morgan Kaufmann

[B17] Domingos P Pazzani MJ Beyond independence: conditions for the optimality of the simple Bayesian classifier International Conference on Machine Learning 1996 105 112

[B18] Kelemen A Zhou H Lawhead P Liang Y Naive Bayesian classifier for microarray data Proceedings of the International Joint Conference on Neural Networks 2003 1769 1773

[B19] Jensen FV Bayesian Networks and Decision Graphs 2001 New York, NY: Springer-Verlag

[B20] Helman P Veroff R Atlas SR Willman C A Bayesian network classification methodology for gene expression data J Comput Biol 2004 11 581 615 15579233 10.1089/cmb.2004.11.581

[B21] Friedman N Linial M Nachman I Pe'er D Using Bayesian networks to analyze expression data J Comput Biol 2000 7 601 620 11108481 10.1089/106652700750050961

[B22] Cooper GF Herskovits E A Bayesian method for the induction of probabilistic networks from data Machine Learning 1992 9 309 347

[B23] Russell SJ Norvig P Artificial Intelligence: A Modern Approach 2002 Upper Saddle River, NJ: Prentice Hall

[B24] Warner B Misra M Understanding neural networks as statistical tools Am Stat 1996 50 284 293

[B25] Ruppert D Wand MP Carroll RJ Semiparametric Regression 2003 Cambridge, UK: Cambridge University Press

[B26] Benjamini Y Hochberg Y Controlling the false discovery rate: a practical and powerful approach to multiple testing J R Stat Soc (B) 1995 57 289 300

[B27] MacKay DJC Probable networks and plausible predictions — A review of practical Bayesian methods for supervised neural networks Network: Computation in Neural Systems 1995 6 469 505

[B28] Schwarz G Estimating the dimension of a model Ann Stat 1978 6 461 464

[B29] Rissanen J Modeling by shortest data description Automatica 1978 14 465 471

[B30] Vapnik V The Nature of Statistical Learning Theory 2000 2 Red Bank: Springer

[B31] Guyon I Weston J Barnhill S Vapnik V Gene selection for cancer classification using support vector machines Machine Learning 2002 46 389 422

[B32] Breiman L Random forests Machine Learning 2001 45 5 22

[B33] Lunetta K Hayward LB Segal J Van Eerdewegh P Screening large-scale association study data: exploiting interactions using random forests BMC Genet 2004 5 5 32 15588316 10.1186/1471-2156-5-32 PMC545646

[B34] Strobl C Boulesteix A-L Kneib T Augustin T Zeileis A Conditional variable importance for random forests BMC Bioinformatics 2008 9 307 18620558 10.1186/1471-2105-9-307 PMC2491635

[B35] Long N Gianola D Rosa GJM Weigel KA Avendano S A marker-assisted assessment of genotype by environment interaction: SNP-mortality association in broilers in two hygiene environments J Anim Sci 2008 86 3358 3366 18765852 10.2527/jas.2008-1021

[B36] Gianola D Perez-Enciso M Toro MA On marker-assisted prediction of genetic value: Beyond the ridge Genetics 2003 163 347 365 12586721 10.1093/genetics/163.1.347 PMC1462425

[B37] Meuwissen THE Hayes BJ Goddard ME Prediction of total genetic value using genome-wide dense marker maps Genetics 2001 157 1819 1829 11290733 10.1093/genetics/157.4.1819 PMC1461589

[B38] Xu S Estimating polygenic effects using markers of the entire genome Genetics 2003 163 789 801 12618414 10.1093/genetics/163.2.789 PMC1462468

